# Opening the *black box* of machine learning in radiology: can the proximity of annotated cases be a way?

**DOI:** 10.1186/s41747-020-00159-0

**Published:** 2020-05-05

**Authors:** Giuseppe Baselli, Marina Codari, Francesco Sardanelli

**Affiliations:** 1 Dipartimento di Elettronica, Informazione e Bioingegneria, Politecnico di Milano, Via Golgi 39, 20133 Milan, Italy; 2 Present Address: Department of Radiology, Stanford University School of Medicine, 300 Pasteur Dr., Stanford, CA 94305 USA; 3 Unit of Radiology, IRCCS Policlinico San Donato, Via Morandi 30, San Donato Milanese, 20097 Italy; 4 Department of Biomedical Sciences for Health, Università degli Studi di Milano, Via Morandi 30, San Donato Milanese, 20097 Italy

**Keywords:** Artificial intelligence, Decision making (computer-assisted), Diagnosis, Machine learning, Radiology

## Abstract

Machine learning (ML) and deep learning (DL) systems, currently employed in medical image analysis, are data-driven models often considered as *black boxes*. However, improved transparency is needed to translate automated decision-making to clinical practice. To this aim, we propose a strategy to open the black box by presenting to the radiologist the annotated cases (ACs) proximal to the current case (CC), making decision rationale and uncertainty more explicit. The ACs, used for training, validation, and testing in supervised methods and for validation and testing in the unsupervised ones, could be provided as support of the ML/DL tool. If the CC is localised in a classification space and proximal ACs are selected by proper metrics, the latter ones could be shown in their original form of images, enriched with annotation to radiologists, thus allowing immediate interpretation of the CC classification. Moreover, the density of ACs in the CC neighbourhood, their image saliency maps, classification confidence, demographics, and clinical information would be available to radiologists. Thus, encrypted information could be transmitted to radiologists, who will know model output (what) and salient image regions (where) enriched by ACs, providing classification rationale (why). Summarising, if a classifier is data-driven, let us make its interpretation data-driven too.

## Key points

- Clinical rules and best practice require diagnosis and therapeutic decision to be transparent and clearly explained.
- Machine/deep learning offers powerful classification and decision tools, though in a hardly explained black box way.
- We propose to present the current case (CC) with training and/or validation data stored in a library of annotated cases (ACs).
- Appropriate metrics in the classification space would yield the distance between the CC and ACs.
- Proximity with similarly classified ACs would confirm high confidence; proximity with diversely classified ACs would indicate low confidence; a CC falling in an uninhabited region would indicate insufficiency of the training process.

## Background

Machine learning (ML) tools and artificial neural networks, the latter nowadays progressing to deep learning (DL), are known to be data-driven models often treated as *black boxes*. They are currently employed in many fields of human life, including healthcare, in particular medical image analysis [1–3].

DL models are characterised by a set of parameters and hyperparameters (*e.g*., network topology and optimisation parameters), which allow to define a non-linear mathematical function that maps input data to target values [4, 5].

During model development, the massive set of parameters are iteratively tuned either to fit an annotated training set (supervised methods) or to achieve optimal clustering performances in a non-annotated one (unsupervised methods), while model hyperparameters parameters are empirically chosen applying grid or random searching strategies on the validation set. Next, the model is tested on the testing set to prove model generalizability. Therefore, DL models are *the indissoluble result all the steps involved in training and validation phases* that include data collection and preparation as well as augmentation and split and training and validation pipeline [4, 6]. Indeed, even freezing model hyperparameters *changing the training dataset results in completely different models*.

This whole process exploits limited or no *a-priori* knowledge about the physical/biological behaviour of the modelled system without being explicitly programmed for a specific task [7]. However, versatility of use and ability to model complex relationship within data are reached through the design of extremely complex models.

DL data-driven approach opposes to internal modelling, which allows to define the mathematical structure of the model based on physiological a-priori knowledge and to parametrise it with few physical/physiological meaningful variables. Indeed, final DL parameters and hyperparameters do not have any meaning other than contributing to high classification performance of trained models.

Not surprisingly, the overall outcome of DL models is rather obscure, apart that it works, that is to say “the proof of the pudding is in the eating”. However, we should admit that data-driven and internal models share many issues concerning the insight of the underlying mechanisms, when real clinical cases are under analysis. Indeed, the needed simplifications and approximations are transparent to few scholars. Moreover, even the most renown and established models in medicine are practically useless if the statistics of biological variability was not included.

Many issues have risen about the use of data-driven black-box classifiers in diagnostic decisions making, such as the possible reduction of physician skills, reliability of digital data, intrinsic uncertainty in medicine and need to open the DL black box [8]. Those concerns involve model real usefulness, reliability, safety and effectiveness in a clinical environment [9, 10].

While clinical standards may be defined to test model safety and effectiveness, model opacity represents an open issue. Indeed, the General Data Protection regulation introduced by the European Union (articles 13, 14, and 15) includes some clauses about the right for all individuals to obtain “meaningful explanation of the logic involved” when automated decision-making takes place [11]. Thus, the development of enabling technologies and/or good practices able to explain the opaque inner working of these algorithms is mandatory to comply with the important principles behind these clauses [12].

We assume that model opacity may be alleviated by enriching the DL outcomes using the information that the model derives from its training and validation dataset in a user-friendly approach, letting radiologists take their final decision with due criticism.

Paradoxically, *the learning strategies of black-box DL models do facilitate this task*. As mentioned above, DL trained models are defined by their architecture and massive set of parameters encrypting the information of the training and validation sets. So, the training/validation sets and the trained models are assumed as being strongly and binomially linked, which bears the non-trivial consequence that also the training/validation data set should be available to users. In our vision, if data is the only prior of a black box model, this should be made transparent in the same way as physical/physiological priors must be stated for internal (alias white-box) models. Nonetheless, we illustrate a transparency principle based on highlighting annotated cases (ACs) proximal to the current case (CC) out of a library linked to the DL model. The basic requirements are as follows (i) to furnish the *library of ACs* (training and/or validation sets), as annex of the trained algorithm; (ii) save the coordinates of the ACs in the classification space, to be used as indexing within the library; (iii) to define a metric in the classification space permitting to univocally define the proximity of ACs to the CC.

In this article, we describe our approach focusing on a specific DL model, namely a convolutional multi-layer neural networks used to perform a binary classification task.

## ML/DL models in radiology

In the last years, several publications have shown the potential of ML/DL applications in medical imaging [5, 13]. The concern is what to do with classifications performed by trained ML/DL models, since they assume that clinical tasks can be solved using sharp decision boundaries (*what* and *where*), though without providing intelligible explanations (*why*). Also from a clinical point of view, the threshold approach and the hypothetico-deductive model have shown several limitations, especially in primary care due to the low prevalence of specific diseases and the extent and poor differentiation of the diagnostic problem space [14]. On the contrary, searching problem space by inductive gathering and triggered routine has emerged as diagnostic strategy for generalist settings [15].

**Fig. 1:**
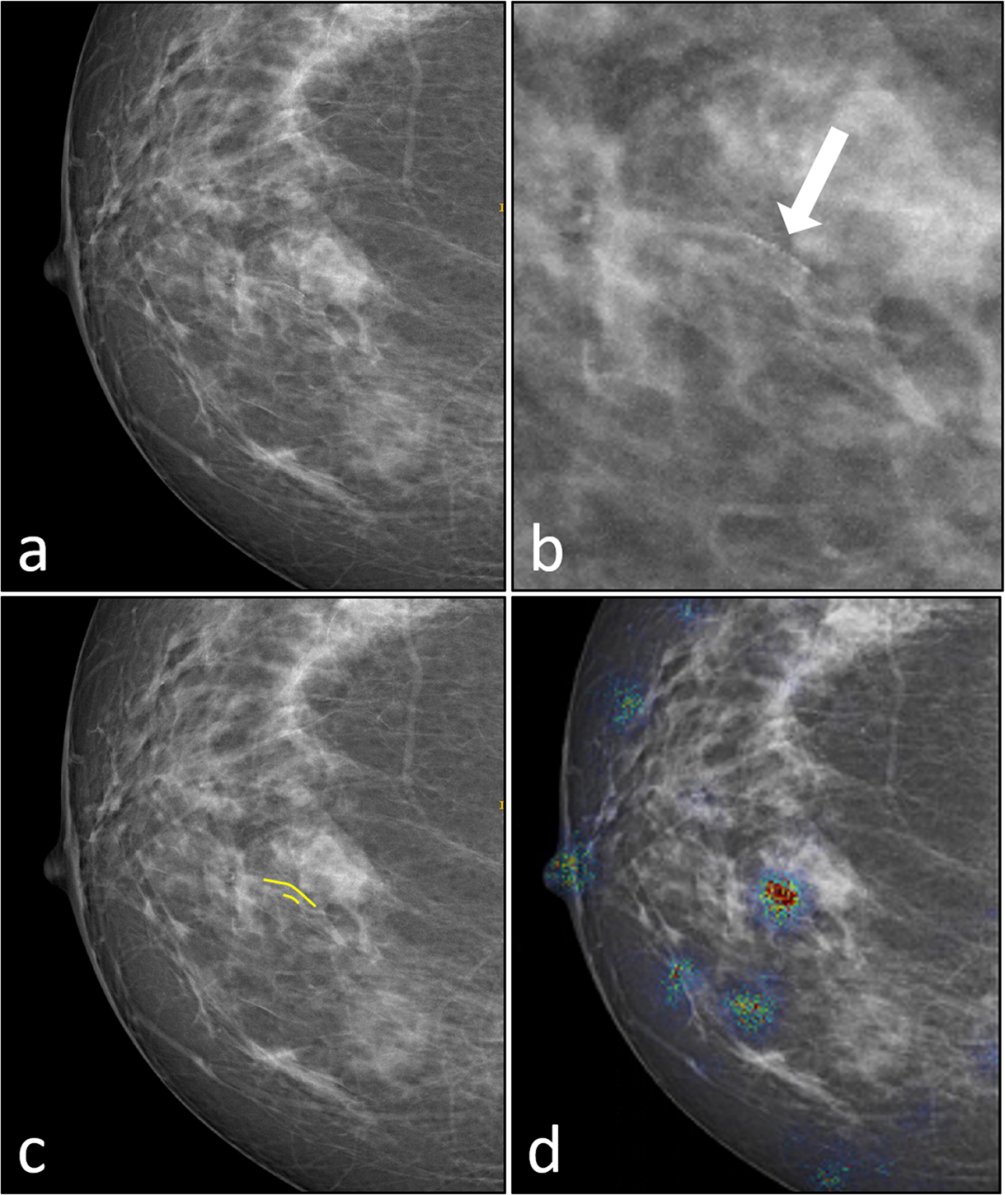
Breast arterial calcifications (BAC) detection by convolutional neural network (CNN). **a** Original image (positive to BAC presence). **b** Detail including the unsegmented BAC (white arrow). **c** Heat map provided by the CNN. **d** Annotated image (BAC in yellow). The heat map (**c**) has the reduced resolution of images input to the CNN

What would skilled radiologists do in the case of diagnostic uncertainty about the CC they have on the screen? Simple, they would search into digital atlases or textbooks cases like the specific one and seek information about the classification confidence of reference cases. Only if they found good similarity and good classification confidence, they would accept the classification proposed by the external source, though with the reported degree of uncertainty. In this light, we propose a DL model outcome inspection strategy that mimics radiologist’s behaviour in a real case scenario. Currently, when complex cases are analysed using DL systems, heatmaps are compared with ground truth annotations to allow the radiologist to trust the black box systems. Figure 1 shows an example of breast arterial calcification (BAC) detection performed using a convolutional neural network (CNN). In the heat map, only the BAC area was above threshold. Manual segmentation (Fig. 1b) is shown for explanatory reasons but was not used in the CNN training: only image level labels (present/absent) were used as ground truth. Note that even after delimiting the BAC area (Fig. 1b), *e.g*., by the heat map (Fig. 1c), the BAC is hardly recognised by a naïf eye. Conversely, the support of the manual segmentation by an expert annotator (Fig. 1d) immediately highlights the searched structure, when back to the original. In our hypothesis, when analysing a CC with no annotation, a surrogate support to decision can be given by similar ACs. Heatmaps are a useful tool to understand which part of the image guided the DL model to its decision but does not provide information about the reason behind it. To better understand the link between that part of the image and the classification outcome, the radiologist must compare it to the ground truth annotation (if available).

However, ground truth annotations are not available while looking at the CC. To give higher support to the final radiologist decision, adding fuzzy or probabilistic information and reference images or cases, should be not difficult. Those solutions could be studied, implemented and validated, in a near future.

## ML/DL uncertainty made explicit: DL example

**Fig. 2:**
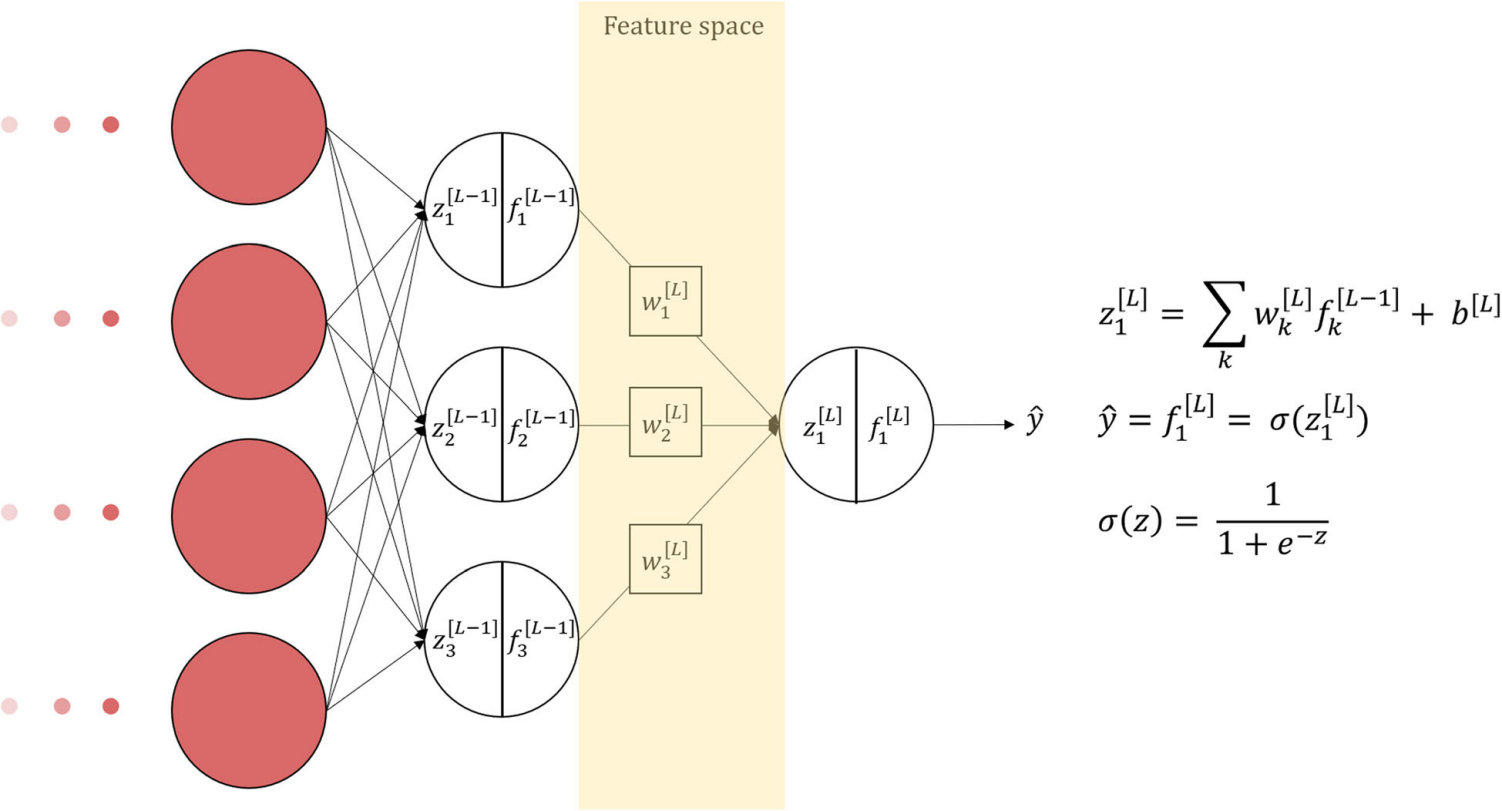
The *L* and *L-1* layers of a deep neural network

Systems based on ML/DL neural networks are complex models composed by a massive number or nodes staked in layers [4]. To have model uncertainty made explicit, a trivial idea can be drawn by observing the classification space. For instance, in DL, this operation can be done by observing  the outputs ${f}_k^{\left[L-1\right]}$ of the last hidden layer, namely the features selected by the previous deeper layers while processing a specific case. The elements of this space do next enter the summation of the output node and, through the nonlinear activation function, provide the sharp classification. In the sake of clarity, the simplest binary classification (either negative or positive), is exemplified in Fig. 2.

**Fig. 3:**
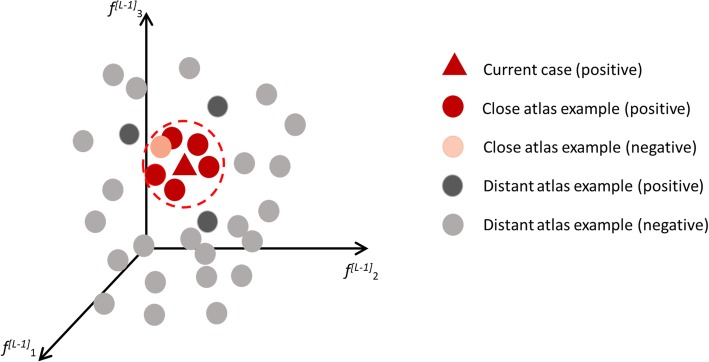
The current case (red triangle) is positioned in the output feature space of *L-1 layer*. A neighbour region (red dashed circle) is fixed and the included training/validation annotated cases (red circles) are considered to provide reference images, classification confidence and ancillary information. Other cases outside the neighbourhood are represented as grey circles

Indeed, elements ${f}_k^{\left[L-1\right]}$are abstract features (alias, meta-features), which result from the passage through many layers that non-linearly combine the meaningful input features. However, they have two important characteristics: (a) validation has recognised them as major determinants of the final decision; (b) they can be put in the classification space of features and their proximity can be assessed by specific metrics, carefully selected among those  available  in the literature [16]. So, the examined case will be a point in this space. Even more importantly, each AC included in the library will find a precise position (fixed and recorded at the end of training or during validation) and those close to the addressed case could be rapidly retrieved through a look-up table. A theoretical example of a CC surrounded by the relevant cluster of libraries ACs is shown in Fig. 3.

## The radiologist entering the black box

The first consequence of the presented approach is that the radiologist would be provided by the pertinent ACs (as by old image atlases).

The second consequence is that the proximal ACs should provide the original images and also ancillary information, such as annotation masks, annotation agreement (alias, human confidence), validation confidence (alias DL confidence), heatmaps localising image regions influencing the classification, subject’s demographics, and clinical profile. The third one is that the distance of the N closest ACs can quantify the density of the library in the region where the current case has fallen, which implies the robustness of training and/or validation specific to the CC.

**Fig. 4:**
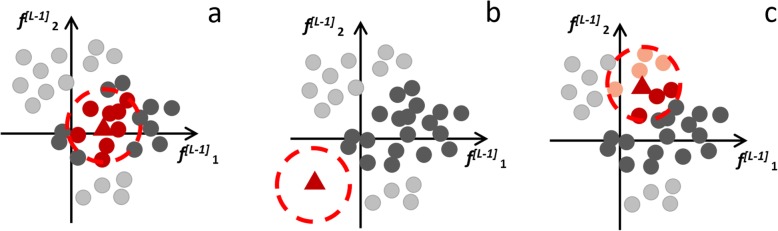
Possible instances of location of the current case (CC) in the feature space. **a** The current case (red triangle) falls into a region crowded with annotated cases (ACs), supposed to be equally classified with high confidence (red circles). **b** The CC falls into an uninhabited region, which would highlight a lack of training or validation similar cases. **c** The CC falls into a crowded region, yet with different classifications of ACs (red and orange circles), most likely with relatively low confidence

Possible instances are shown in Fig. 4: (a) the CC falls into a crowded region with high levels of consensus, which would support the automated classification and also explain it by the CC similarity to homogenous ACs; (b) the CC falls into an uninhabited region, which would highlight a lack of training and/or validation cases similar to the CC; (c) the CC falls into a crowded area, yet with differently classified ACs, most likely in a boundary region with low confidence scores, which uncertainty can be legitimately transferred to the CC classification.

## Transparency and communication barriers

**Fig. 5:**
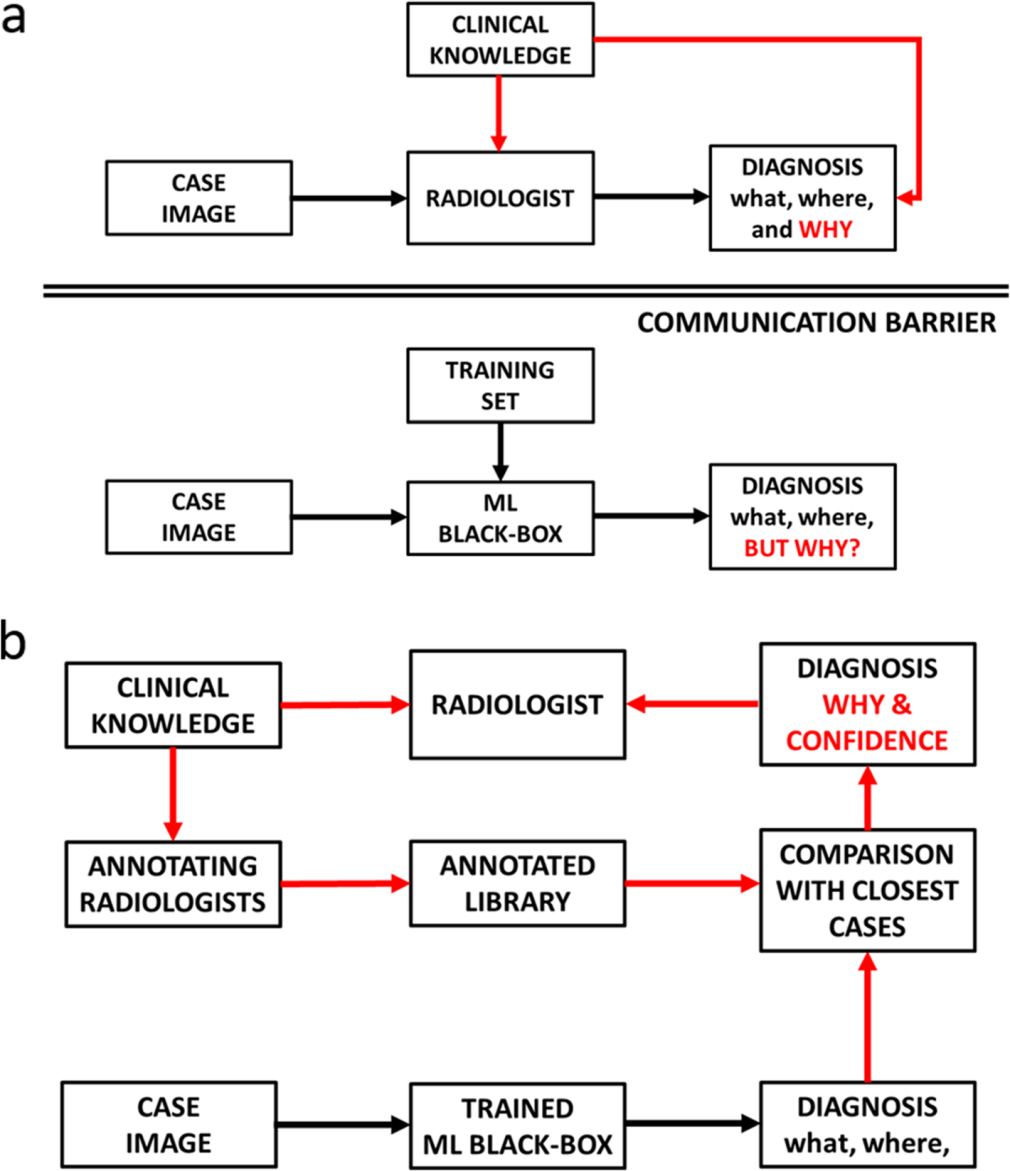
Schemes of the diagnostic process aided by machine learning tools to show process differences with (**a**) and without (**b**) communication barriers. The second option allows the clinician to retrieve information about classification results (*what*), object localisation (*where*), and added information on the decision-making process (*why*) derived from the annotated library

It is worth emphasising that the annotation process exploited for training, validation, and testing in ML/DL models implies that significant clinical knowledge and rating efforts were exploited by the model developers and are ultimately encrypted inside the trained model parameters. However, as shown in Fig. 5a, this is not transmitted to the clinician in charge to the CC, who must rely on its own experience in order to justify the model prediction. Hence, a communication barrier is cast, even if the whole process from development to application subtends common clinical knowledge and classification rule consensus. Conversely, the abovementioned information is better conveyed by means of ACs from the library. *The part of library relevant to the CC is time to time activated based on a proximity concept*. Hence, the user radiologist will benefit not only of the classification (*what*) and localisation capabilities (*where*) of the model, but also will have reference cases permitting to explain the decision (*why*) and assess its confidence (Fig. 5b). Furthermore, this process would help in detecting cases poorly addressed by the model, thus permitting to give feedbacks to developers, and to  allow those feedbacks  to be collected, verified and applied for improved model versions  prior to being certified and delivered to the clinical community  as a new improved release. Medicine has been often improved via empirical observations shared to the clinical community. Also, ideas for  new research  projects frequently arise from empirical, anecdotal observations. A black-box application of DL approaches could interrupt this virtuous-loop. Our hypothesis may facilitate comprehension of the developers’ view to users (feedforward) as well as give back to developers the users observations (feedback). Nothing new, as is in many arts and in medicine.

Additional information provided by our solution may cause a reporting time increment. However, a close inspection of similar cases should not be done on regular basis. It should be performed mainly for critical cases and/or in order to pinpoint systematic classification flaws and for DL algorithm  debugging (*e.g*., to enrich a class poorly represented in the training and validation sets). Moreover, more information about system decision may be provided on demand when needed.

Conversely, we foresee that the most practical outcome to clinical decision support would be to provide objective and well-explained indexes of classification confidence specific to the CC such as the  density of the proximal classification space with similar cases. We believe that this approach will provide a significant added value to existing solutions allowing a more tailored analysis of DL outcomes compared to the indexes of the classifier performance, which give overall statistics.

## Conclusions

We are currently impressed by the emerging role of ML/DL in medicine and radiology. More and more, computer algorithms are shown to outperform radiologists, exploiting curiosity and fears of downsizing of professional roles. However, *the patients’ interest is not to know whether a ML/DL tool is better than a physician but if a radiologist with an ML/DL aid is better than the same radiologists without*.

The way to open the back box we presented here can favour an interactive cooperation between radiologists and automated systems, soliciting the radiologists’ (biological!) neural networks to integrate their previous clinical experience by visualising well-labelled cases that the system has classified as proximal to the CC, so allowing for a critical assessment of the performance of the automatic tool. Moreover, it may serve as a valuable tool to test the generalizability of the proposed model during development and certification processes. This perspective offers a novel *paradigm of proximity for ML/DL transparency*. However, we did not tackle problems such as the dimensional reduction of the classification space to few weighty meta-features and the choice of the most effective metrics within. We hope that these non-trivial methodological problems might solicit brilliant minds in the field to experiment the best implementation ways.

## Data Availability

Not applicable

## Abbreviations

AC: Annotated case
BAC: Breast arterial calcification
CC: Current case
CNN: Convolutional neural network
DL: Deep learning
ML: Machine learning
